# 

**Published:** 1884-06

**Authors:** 


					﻿The Fifth Annual Meeting of the I. H. A.—The Fifth annual
meeting of the International Hahnemannian Association will be held on Friday
;and Saturday, June 13th and 14th, 1884, in Washington, D. C., in the parlors
of C. Pearson, M. D., No. 611 Twelfth Street, Northwest, commencing at ten
i©’clock A. m., Friday.	Geo. F. Foote, M. D., President.
NOTICE.
All articles for publication, books for review, business communications, etc.,
must be sent to'^Editor, 2109 Chestnut Street, Philadelphia.
				

